# Frameworks for Community Impact - Community Case Study

**DOI:** 10.3389/fpubh.2020.00197

**Published:** 2020-06-02

**Authors:** Laural Ruggles

**Affiliations:** Northeastern Vermont Regional Hospital, St. Johnbury, VT, United States

**Keywords:** community benefit, accountable health community, community health needs assessment, non-profit hospital, social determinansts of health

## Abstract

The Affordable Care Act of 2008 placed specific community health needs assessment and community benefit reporting requirements on US not-for-profit hospitals. The requirements are straightforward, but come with no expectation for synergy between the needs assessment and the community benefit spending, no direction on how to design systems to improve community health, and with surprisingly little accountability for improving health outcomes. With the help of diverse community partners, one Critical Access hospital in rural Vermont has successfully linked the needs assessment with community benefit dollars to address upstream contributors of health. In 2014, Northeastern Vermont Regional Hospital lead the creation of NEK Prosper: Caledonia and Southern Essex Accountable Health Community with a mission to tackle poverty as the ultimate root cause of poor health in the region. This article outlines how a hospital community health needs assessment ignited a change in how community partners worked together, aligned organizational strategies, and overcame industry jargon barriers to create regional system change to improve health. And how that same hospital has used community benefit dollars to accelerate action at the community level.

## Introduction

This article outlines how Northeastern Vermont Regional Hospital (NVRH) is able to use its community health needs assessment as both a catalyst to change how community partners work together, and to inform how best to spend the hospital community benefit dollars to impact community health.

NVRH is a 25 bed Critical Access Hospital in northern Vermont. The 2012 NVRH Community Health Needs Assessment (CHNA) identified poverty as one of the top health priority areas. Poverty, and the symptoms of poverty like inadequate access to healthcare, healthy food, transportation, and education, is a well-documented root cause of poor health (1, 2). Low income adults are more likely to suffer difficulties in their daily lives due to chronic illness, while children living in poverty are often left with risk factors that can affect their health throughout their lives (3) Consequently, tackling poverty in the hospital service area became a priority issue for NVRH and its leaders, particularly the CEO. As part of the 2012 CHNA implementation plan, NVRH committed to convene community leaders to address the issue of poverty as the upstream, systemic driver of poor health and health inequity.

Convened by invitation of NVRH, the leaders of the regional Federally Qualified Health Center (FQHC) and home health agency, designated mental health agency, community action agency, council on aging, and designated regional housing organization began meeting regularly at the hospital. As they talked about what they each could do to address poverty and researched how they could work better together in a strategic and collaborative way, two models emerged: The Accountable Health Community (AHC) and collective impact (CI) models.

AHC is an emerging model gaining popularity across the US. An AHC is responsible for the health and well-being of everyone who lives in a geographic region. The AHC model recognizes that the health of a population is determined by multiple factors: healthcare, environment, socio-economic status, and individual behaviors. (4) The Center for Medicare and Medicaid Services (CMS) says the AHC model “addresses a critical gap between clinical care and community services in the current health care delivery system” (5).

The Prevention Institute has embraced the AHC model as a “promising vehicle toward reaching the full potential of the Triple Aim.” The Prevention Institute has identified nine core elements of the AHC model: multi-sectoral partnership; integrator organization; governance; data; strategy and implementation; community engagement; communications; and sustainable financing (6).

The AHC model outlines one structure to foster collaboration (7). True collaboration requires multi-sector partners work well-together (8). This is where the collective impact model can help.

CI has been articulated as a method for solving large scale social problems by “a systemic approach to social impact that focuses on the relationships between organizations and the progress toward shared objectives.” Successful CI initiatives have five conditions that together produce true alignment and lead to powerful results: a common agenda, shared measurement systems, mutually reinforcing activities, continuous communication, and backbone support organizations (9).

## Context

NVRH is located in Vermont's Northeast Kingdom, a region known for its rugged rural landscape and independent and spirited people. The primary service area for NVRH is Caledonia and southern Essex counties, with just under 30,000 people. Population density in Caledonia County is 48.1 persons per square mile and 9.5 persons per square mile in Essex County (10). Both counties are bordered by the Connecticut River and New Hampshire to the east.

The 25 bed hospital is the largest employer in the region, with over 600 employees. The hospital operates four rural health clinics and six specialty medical offices. A different entity operates three FQHC's and home health and hospice for the region. All the primary care offices in the region are recognized NCQA Patient Centered Medical Homes. Mental health services are provided by a regional designated mental health agency and many independent providers in private practice. There are several independent long-term care facilities in the area. Comprehensive cancer care services are located on the NVRH campus, but provided by the nearest (70 miles to the south) tertiary center. A private for-profit dialysis center provides services in a building owned by NVRH. Medically Assisted Treatment (MAT) for opioid addiction is provided by a private for-profit organization located down the road from NVRH. The mental health agency and several primary care offices operated by the hospital and the FQHC also provide MAT services.

NVRH has a long history and reputation for working collaboratively and embracing the idea that health happens outside the walls of the hospital. Over the decades, NVRH leadership has spearheaded the formation of prevention coalitions to address obesity and substance use, providing staff resources, meeting space, and funding for coalition initiatives. Both NVRH leadership and staff routinely works with local and state public health staff though the Vermont Department of Health on prevention and public health initiatives driven by the Vermont State Health Improvement Plan, and data like the Behavioral Risk Factor Surveillance Survey and the Youth Behavior Risk Survey collected by the Health Department, as well as local Department of Health priorities.

The NVRH service area was the first of two pilot communities funded by the Vermont Blueprint for Health in 2005, and the first Integrated Medical Home and Community Health Team pilot community created under Act 71 (11). The robust and active Blueprint for Health Community Health Team continues to provide a forum for coordinated care between direct service providers from healthcare, human services, and community-based organizations.

## NEK Prosper!

In 2014, NVRH lead the creation of NEK Prosper: Caledonia and Southern Essex Accountable Health Community with a mission to tackle poverty as the ultimate root cause of poor health in the region.

That initial informal group of community leaders convened by NVRH in 2014 has since added the state-wide foodbank and the regional United Way and become the leadership team. NEK Prosper has provided the forum for the leadership team decision makers come together to strategically align their organizations, something that did not happen prior to the formation of the AHC.

The leadership team embraced the frameworks provided by the AHC and CI models. There is a formal governance and decision-making structure, shared measures for success, and intentional methods for community engagement. Basic meeting etiquette and equitable participation is ensured by reviewing standard norms of behavior, such as “listening with intent” and “address issues directly and succinctly” at each meeting. Leadership team members have adopted norms of behavior for meetings. They have all signed a memorandum of understanding (MOU) that outlines the mission and purpose of the AHC, specific roles and responsibilities of the leadership team members, and a process for decision-making. Stewardship has been an important guiding principle for all members; the advice to “wear two hats – those of your organization and this partnership” is included in the norms of behavior.

**Figure 1:**
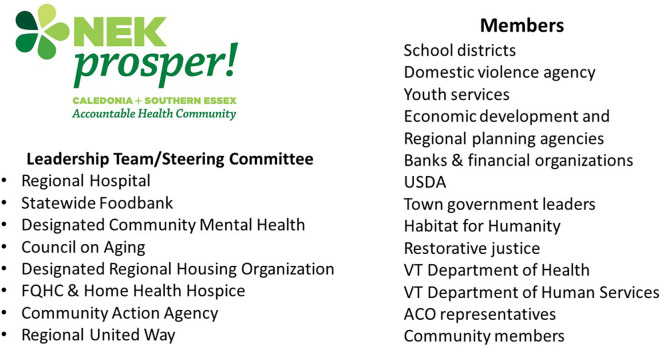
Member Organizations for NEK Prosper.

Today, the AHC includes members from healthcare, human services, housing, transportation, mental health, community action, charitable food, funders, school districts, domestic violence agency, youth services, economic development and regional planning, banks/financial organizations, town government, restorative justice, and State agencies including Vermont Department of Health and Vermont Department of Human Services (Figure 1). All NEK Prosper members sign a culture statement that encourages innovative thinking, sharing of resources, and working relationships based on trust and respect. Rather than creating another needs assessment, NEK Prosper officially adopted the hospital community health needs assessment (CHNA) as the official community needs assessment of the AHC in 2016.

The name NEK Prosper: Caledonia and Southern Essex Accountable Health Community was officially adopted in 2018. NVRH serves as the backbone organization for NEK Prosper.

Workgroups called Collaborative Action Networks (CANs) include community member participation and focus on each of NEK Prosper's five outcome areas: our community will be well–nourished, well-housed, physically healthy, mentally healthy, and financially secure. NEK Prosper and the CANs use Results Based Accountability™ to measure impact on health.

## Programmatic Elements

The purpose of the NVRH community health needs assessment is to identify initiatives at the individual, community, environmental, and policy level, as well as programs and services that meet the hospital's mission to improve the health of people in the communities it serves. When it came time for NVRH to complete the 2018 CHNA, the leading criterion for setting community health priorities was the ability to work within the NEK Prosper framework to best capitalize on existing community resources and assets.

The 2018 CHNA built on the foundation of the previous assessments. New for 2018, the CHNA used the framework of NEK Prosper. Additionally, the CHNA was advised by the data compiled and the community engagement work already done by NEK Prosper, and adopted the mission of the NEK Prosper to reduce poverty in the region.

The CHNA data collection identified low-income families, and older adults as the most vulnerable population. The CHNA validated the objectives of NEK Prosper that communities will be financially secure, physically healthy, mentally healthy, well-nourished, and well-housed. Consequently, the NVRH 2018 CHNA proposed that **over the next three years, NVRH will implement initiatives, and programs and services that work to meet these five objectives to improve health in the community, while intentionally addressing the underlying causes of health disparities**.

Like NEK Prosper, the 2018 NVRH CHNA Implementation Plan and Evaluation use Results Based Accountability™ (RBA) to measure impact, evaluate initiatives, and drive action and change. RBA provides a step by step process to get results. RBA defines both population level (whether we have achieved goals for a defined population) and performance level (how well a program or service is working) measures. (12).

In fiscal year 2019, with an intentional effort to use community benefit dollars to accelerate action, the hospital budgeted $93,000 from operations to fund initiatives of the five CANs of NEK Prosper. The initiatives and dollar amounts are outlined in the CHNA Implementation Plan. For that first year, not all the CANs had initiatives ready for funding. The same amounts were budgeted for fiscal year 2020.

The CANs are data driven and use a common template and tools to decide which community strategies to implement. The Well-Nourished CAN launched the Food Hero Social Marketing Campaign in May 2019. Food Hero is a program from Oregon State University Extension Service with funding from SNAP-ED. The goal of the program is to increase fruit and vegetable consumption by creating and disseminating low cost, easy to prepare, and healthy recipes (13).

NVRH Community Benefit dollars purchased re-useable grocery bags with the Food Hero and NVRH logo. The bags are distributed at events sponsored by NVRH and the partner organizations of the Well-Nourished CAN. Large Food Hero banners attract attention at local events. Food Hero themed placemats are used at the hospital and senior meal sites.

Using Results Based Accountability™ (RBA) principles, the Well-Nourished CAN tracks the number of sites using Food Hero materials, social media engagements, and the number of Food Hero recipes distributed to measure performance level results. The CAN will use a Food Hero qualitative evaluation tool to measure behavior change in spring 2020. The CAN uses population level indicators collected by the Vermont Department of Health (fruit and vegetable consumption and the prevalence of hypertension) to measure long term impact.

Other CAN initiatives funded by NVRH community benefit dollars are the popular smoothie bikes for use at school and community events as part of the Physically Healthy CAN's community-based campaign to increase physical activity, and stipends for fitness providers to offer free pop up fitness classes in local parks. NVRH has funded a small pilot project that pays for complimentary therapies like acupuncture for people coping with mental health issues under the direction of the Mentally Healthy CAN. Every CAN initiative is evaluated for impact by using RBA performance measures of “how much, how well, and is anyone better off.” Each CAN measures community wide impact by using population level indicators such as percentage of people getting the recommended amount of physical activity or regional rates of suicide. Impact dashboard for some of the CANs can found at the NEK Prosper website.

The partners in NEK Prosper are not stopping with community-based interventions of the CANs. Two wellness funds were created thanks to the strong culture of stewardship, and leaders focused on action to improve health by tackling poverty.

Working with local economic development and financial partners NEK Prosper members are ready to launch the NEK Prosperity Fund using a capital stacking approach to raise funds to free up almost a million dollars currently held by the regional Community Development Financial Institution (CDFI) to invest in small and emerging local businesses. The purpose of the fund is to act as an investment vehicle aimed at promoting economic development by offering loans to local businesses, as well as supporting the overall well-being of the employees and customers of the business. Loans will be given to businesses that might not qualify for traditional bank loans or other loans offered by the CDFI. These more “at risk” businesses will need a high level of technical assistance to be successful. An Advisory Committee of NEK Prosper will assist the CDFI in developing general policies for mission driven funding.

The NEK Prosper leadership team has committed to raising $200,000 for a loan loss reserve fund to protect the original capital loan fund assets, and to pay for additional business support services by the experienced CDFI staff. It is expected the money will be raised quickly with investments from the hospital, other leadership team organizations, and local businesses.

In spring 2020, NEK Prosper launched the Healthy Cents Fund. The Healthy Cents Fund is available for local organizations for innovative upstream interventions or investments that will create healthy and thriving communities and positive social, economic, or environmental impact. The fund aims to accelerate the work of NEK Prosper and move the AHC closer to the five outcome areas. The value-based payment environment was key to the creation of this fund. Funding for the Healthy Cents Fund comes from Medicaid capitated payments to NVRH paid through the Vermont All Payer Model and the state-wide Accountable Care Organization. Rather than wait for potential shared savings, NVRH takes 1% off the top of the per member per month capitated payments to finance the Healthy Cents Fund, or about $58,000 annually.

Both funds require community engagement activities, and must tie directly to the five outcomes areas of NEK Prosper and the health priorities of the CHNA. Social return on investment is measured using a modified logic model table linking funded activities to short and long term social outcomes.

## Discussion

In a value-based payment environment, hospitals have the financial incentives to keep people well and out of the hospital *and* the flexibility to use hospital resources to address the social determinants of health. It is time for hospitals to put resources into prevention and the social and environmental factors that make people sick (1). Nationally, researchers and policy makers are looking for ways for hospitals and partners to combine resources in a more systemic way (14, 15).

Hospitals already have a tool in place to identify the community needs and priorities: the CHNA. The data and community input gathered during the CHNA process provides the roadmap for where hospitals can best invest resources to make the most impact on health. The community benefit requirements of the Affordable Care Act make it possible for hospitals to get credit for their investments. Yet, few hospitals are investing in “community building” projects that address social determinants (2). Additionally, there is clear consensus that a comprehensive approach to improving health requires multisector partners working in sync. However, we are falling short of all we can do to truly improve health and well-being (8).

NVRH and the partner organizations in the region have used the promising models of collaboration of Accountable Health Community (AHC) and collective impact (CI) to align their strategies, organization resources, and funding. The models provide the structure to work collaboratively, while holding people accountable for their contributions to the goals of NEK Prosper. AHC and CI have helped create an atmosphere of trust and a process for measuring results.

Leadership from the hospital CEO and the executive directors of other community agencies was a critical component to initiating and continuing the collaboration. Stewardship and trust are two additional elements that are essential. The CI model provides a model for identifying and incorporating these elements into concrete activities.

The role of the hospital was critical to the success of NEK Prosper. Despite its small size, NVRH is a leading force in the community. Additionally, the financial contribution provided through the community benefit funds enabled concrete actions that the community might otherwise have struggled to achieve.

Lessons Learned and Tips for Success:

- Don't reinvent the wheel. Use existing models and frameworks to create a community collaborative structure the works in your community.
- Be strategic in making your list of who needs to be at the table. Include traditional health and human service partners, community-based organizations focused on social determinants, local and state government, funders, and less traditional partners like for-profit business and economic development agencies.
- Finding common ground with less traditional partners may take some time; expect communication barriers. NVRH and NEK Prosper found that banks and economic development agencies wanted the same thing – a healthy prosperous community; however, industry specific jargon made it difficult to identify common goals. Engage these partners in your work by asking for their expertise in finding financial resources for projects and measuring financial return on investment. In exchange, health and human services can offer expertise in social return on investment measures, and provide specific services and programs to improve the health and well-being for employees of these partners and for the employers and customers they work with every day.

We inherently know that we are better together, stronger together, and can accomplish more together. Using current partnership frameworks like Accountable Health Community and collective impact, hospitals can provide the data - CHNA, the funding - community benefits, and the leadership to foster a culture of stewardship to truly create and maintain healthy communities.

## Conclusions

As hospitals work to improve health in their communities, they must be intentional about improving the systems and structures within their organizations and regions to support health, well-being, and equal opportunities for all.

The recipe for success includes a strong foundation built on three models: Accountable Health Community, Collective Impact, and Results Based Accountability™ to guide operations, keep community partners heading in the same strategic direction, and quantify and measure results. Adding three key ingredients: leadership, stewardship, and action to the foundational structure drives NEK Prosper toward high impact and a healthier, and potentially more prosperous, region.

## Data Availability Statement

The datasets generated for this study are available on request to the corresponding author.

## Author's Note

This is a community case study as described by one author. The author has a unique perspective because of her involvement in the initial and ongoing operations of NEK Prosper. She is also the staff person at NVRH in charge of both the CHNA process and the community benefit reporting.

## Author Contributions

The author confirms being the sole contributor of this work and has approved it for publication.

## Conflict of Interest

The author declares that the research was conducted in the absence of any commercial or financial relationships that could be construed as a potential conflict of interest.
